# Usability and acceptability of self-testing for hepatitis C virus exposure in a high-prevalence urban informal settlement in Karachi, Pakistan

**DOI:** 10.1186/s12879-024-09925-6

**Published:** 2024-09-27

**Authors:** Sara Mazzilli, Muhammad K. Aslam, Javed Akhtar, Marta Miazek, Yves Wailly, Saeed Hamid, Sonjelle Shilton, Dimitri Donchuk, William A. de Glanville, Petros Isaakidis

**Affiliations:** 1 Scuola Normale Superiore, Pisa, Italy; 2 Médecins Sans Frontières, Operational Centre Brussels (MSF OCB), Karachi, Pakistan; 3 Médecins Sans Frontières, Operational Centre Brussels (MSF OCB), Brussels, Belgium; 4 Department of Medicine and Director of the Clinical Trials Unitat , The Aga Khan University, Karachi, Pakistan; 5 FIND, Geneva, Switzerland; 6 Médecins Sans Frontières, Southern Africa Medical Unit (SAMU), Johannesburg, South Africa; 7 Present Address: School of Biodiversity, College of Medical, Veterinary and Life Sciences, One Health and Veterinary Medicine, University of Glasgow, Glasgow, UK; 8 Present Address: Clinical and Molecular Epidemiology Unit, Department of Hygiene and Epidemiology, University of Ioannina School of Medicine, Ioannina, Greece

**Keywords:** Hepatitis C virus, HCV, Self-test, HCVST, Usability, Acceptability

## Abstract

**Background:**

Hepatitis C virus (HCV) antibody self-testing (HCVST) may help expand screening access and support HCV elimination efforts. Despite potential benefits, HCVST is not currently implemented in Pakistan. This study aimed to assess the usability and acceptability of HCVST in a high HCV prevalence informal settlement in Karachi, Pakistan.

**Methods:**

We performed a cross-sectional study in a hepatitis C clinic from April through June 2023. Participants were invited to perform a saliva-based HCVST (OraSure Technologies, USA) while following pictorial instructions. A study member evaluated test performance using a standardized checklist and provided verbal support if a step could not be completed. Perceived usability and acceptability were assessed using a semi-structured questionnaire. The HCVST was considered successful if the participant was able to complete all steps and correctly interpret test results. Overall concordance and positive and negative agreement were estimated in comparison with the HCVST result read by the study member (inter-reader concordance and agreement) and result of a second rapid HCV test (Abbott Diagnostics Korea Inc, South Korea) performed by a trained user (inter-operator concordance and agreement).

**Results:**

The study included 295 participants of which 97 (32%) were illiterate. In total, 280 (95%, 95% CI 92–97%) HCVSTs were successful. Overall, 38 (13%) people performed the HCVST without verbal assistance, 67 (23%) needed verbal assistance in one step, 190 (64%) in two or more. Assistance was most often needed in managing the test buffer and test reading times. The inter-reader concordance was 96% and inter-operator concordance 93%. Inter-reader and inter-operator positive percent agreement were 84 and 70%, respectively. All participants reported they would use HCVST again and would recommend it to friends and family.

**Conclusion:**

Saliva-based HCVST was very well accepted in this clinic-based setting. However, many people requested verbal support in several steps, highlighting the need for clear instructions for use and test devices that are simple to use, particularly in low literacy settings. Moderately low positive percent agreement with the results of a rapid test performed by a trained user highlights potential uncertainty in the accuracy of HCVST in the hands of lay users.

**Supplementary Information:**

The online version contains supplementary material available at 10.1186/s12879-024-09925-6.

## Introduction

Hepatitis C virus (HCV) is a major cause of chronic liver disease globally, affecting about 58 million people, particularly in low- and middle-income countries (LMICs) [1]. The advent of curative, short-duration, pan-genotypic direct-acting antiviral (DAA) therapy has transformed HCV treatment, making it universally accessible and affordable in many LMICs [2]. In 2016, the World Health Organization (WHO) launched the Global Health Sector Strategy on Hepatitis 2016–2021, targeting the elimination of HCV as a public health threat by 2030 [3].

In line with this strategy, Pakistan announced the “Prime Minister’s Programme for Hepatitis” to eliminate hepatitis C (and B) from the country by the end of the decade [4]. Pakistan is considered to have the second highest prevalence of HCV infection in the world, with an estimated six million people in need of treatment [5]. To find and treat these chronically HCV-infected people, an enormous expansion of testing is required.

It has been estimated that only 20% of people living with HCV in low-income countries know their status [6]. New strategies to increase HCV testing coverage are urgently needed to reach chronically infected people and achieve the target of hepatitis elimination by 2030. Self-testing, where people collect their own specimen, perform a simple rapid test, and interpret the result, has been recommended by WHO since 2016 [7] as an accurate, safe, and acceptable approach to reach people with human immunodeficiency virus (HIV) who may not otherwise access testing [8]. There are limited examples of self-testing for infectious disease in Pakistan but a pilot project in Larkana demonstrated that distributing HIV self-testing (HIVST) kits to transgender individuals was an acceptable approach within this key population [9]. The widely documented success of HIVST globally in increasing the uptake of HIV testing among hard-to-reach groups [10–12] has led the WHO to strongly endorse HCV self-testing (HCVST) in settings where appropriate linkage to care can be provided [13].

At the time of the study, there were no products approved by a stringent regulatory authority in any country or prequalified by WHO as HCVST. However, several professional-use HCV RDTs already prequalified by WHO could potentially be adapted for self-testing use. The current reported experience with HCVST remains limited to a few countries and a few high-risk populations [14–22]. A study to assess the acceptability and impact of home delivery of the HCV self-test in the context of a house-to-house HCV micro-elimination programme in Pakistan was launched in 2021 in Karachi [23] but findings are not yet published. There is a need for a series of evaluation studies investigating the acceptability and usability of HCVST in a range of HCV-prevalent settings to guide further scale-up. These evaluations should include marginalised populations such as those living in informal settlements. Such populations typically have reduced access to formal health care and often have high prevalence of HCV infection [25] and may therefore particularly benefit from HCVST. However, levels of literacy are often low in such settings, raising questions about the usability and acceptability of unsupervised HCVST.

This study aimed to assess the usability and acceptability of a saliva-based HCVST in a sample of people attending a hepatitis C clinic in Machar Colony, one of the largest and oldest informal settlements in Karachi, Pakistan.

## Materials and methods

### Study setting

The study was conducted at a Médecins sans Frontières (MSF) clinic offering free of cost screening and treatment for hepatitis C located in Machar Colony. This is an unplanned settlement in Karachi with an estimated 150,434 residents, constituting a population that includes many migrants and their descendants from across Pakistan as well as from Afghanistan, Bangladesh and Myanmar [24]. A recent prevalence study reported an HCV-seroprevalence of 12.4% and a viraemic prevalence of 4.0% [25]. The seroprevalence in this informal settlement was more than double the reported national average for Pakistan [5].

### Study design

This was a cross-sectional study. New patients attending the MSF clinic for HCV screening from 26 April through 22 June 2023 aged 18 or above were invited to participate. Recruitment followed the flow of patients attending the walk-in clinic, with people invited to participate when a dedicated study staff member had completed the data collection procedures with the previous participant. Adults who were unable to provide informed consent due to severe mental conditions that impaired decision-making were excluded. To avoid biasing results by known serostatus, individuals who reported previously testing positive on HCV rapid diagnostic test (RDT) or HCV polymerase chain reaction (PCR) were excluded from study participation. The people excluded from the study were provided with free of cost HCV screening and clinical management following standard clinic protocols. No compensation or other incentives were given to study participants.

### Study procedures

For the self-test, the OraQuick HCV assay (OraSure Technologies, Bethlehem, PA, USA) detecting antibodies in a saliva sample was used. This test has WHO pre-qualification for professional use on whole blood and other body fluids, including saliva.

Study participants were invited to a dedicated room in the clinic where self-testing could be done in private. Testing was overseen by a study member, an experienced laboratory technician who remained in the room throughout the HCVST procedure. Study participants were shown a standardised set of pictorial instructions for use (IFU) complemented by short sentences in Urdu, the national language (Supplemental material Fig. 1 and Fig. 2). Excluding the addition of the Urdu text by the study team, these IFU followed the same structure as the simplified IFU provided by the test manufacturer and used the same images for the testing procedure. A short, standardised verbal description of the RDT procedure was provided by a laboratory technician that followed key steps in the IFU. The participant was then invited to perform the test by following the IFU. The laboratory technician remained silent during the procedure with the participant encouraged to follow the IFU rather than ask questions. The participant could make mistakes provided these did not have implications for their safety. If the participant reported that they could not complete any stage themselves, they first were offered verbal assistance by the laboratory technician, the nature of which was recorded. If they were still unable to complete the stage, the self-testing procedure was considered to have ended.

**Fig. 1:**
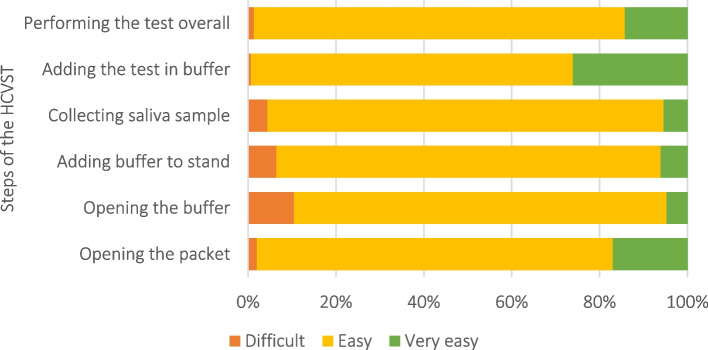
Perceptions of HCV self-test usability at different steps as assessed among 295 study participants attending a hepatitis C clinic in Karachi, Pakistan, April-June 2023

After the completion of the HCVST, participants were asked to read the result of the test they had performed. The test result was re-read by the laboratory technician who had observed the procedure. To confirm results of the HCVST, an SD Bioline (Abbott Diagnostics Korea Inc, South Korea) RDT was conducted by the laboratory technician using a finger prick sample. The technician reading the results of this test was not blinded to the results of the HCVST. The SD Bioline RDT was the standard screening assay used in the MSF clinic and was therefore used for this comparison rather than an additional saliva based OraQuick HCV assay. This ensured participants received the same standard of care as all other people attending the clinic. Hepatitis C treatment was offered free of charge following standard clinic protocols to all study participants who were found to be infected with HCV on confirmatory PCR.

### Data collection

Data were collected on a mobile phone in a dedicated Kobocollect form (www.kobotoolbox.org). The study technician observing the HCVST recorded completion of each step following a standard checklist (Supplemental material Annex 1). Mistakes made, difficulties observed, and any verbal or direct help provided to the participant were recorded. While waiting for their test results, patient demographic data and overall perception of HCVST were collected. Literacy was assessed by asking the participant to read the sentence ''Today we are testing for a liver disease that can affect people of any age'' written in the national language, Urdu. Evaluation of acceptability of HCVST included participant confidence in performing the test by themselves in their own homes, their willingness to recommend HCVST to friends and family members and their preference for blood- versus saliva-based HCVST.

### Data analysis

The self-testing procedure was considered successful if each of the following criteria were met: 1. The test was valid as indicated by the presence of a clear control line; 2. The participant correctly interpreted the test result; and 3. The participant was able to complete all steps in the standardised checklist without direct assistance (excluding verbal assistance). The proportion of people successfully completing HCVST gave the ‘success ratio’.

Inter-reader concordance was calculated as the percentage agreement between the results of the HCVST as interpreted by the participant and the results of the same test interpreted by the laboratory technician. The percentage agreement between positive results (positive percent agreement) and negative results (negative percent agreement) were also calculated. Inter-operator concordance was calculated as the percentage agreement between the HCVST result reported by the participant and the result of the confirmatory test conducted by the laboratory technician. Positive and negative percent agreement were also calculated for this comparison. Cohen’s kappa coefficient [26] was calculated for both concordance measures.

Descriptive statistics for the characteristics of people performing the supervised HCVST were generated. Descriptive summaries of people’s assessment of the ease of use of the test, willingness to repeat the test on their own in the future and reported preference of saliva- versus blood-based tests were derived.

Analysis was conducted in R statistical environment version 4.1.1.

### Sample size estimates

A systematic review reported agreement for unsupervised HIV self-testing ranging between 80 and 100% when compared to a test performed by a health worker, with a pooled agreement of 97% [27]. We estimated a sample size that would ensure the lower bound of a one-sided 95% exact binomial confidence interval for the expected success ratio would be greater than 80% on at least 80% of occasions on the assumption of a minimum success ratio of 87%. This resulted in a minimum sample of 200 study participants. This sample size was estimated in R statistical environment based on simulation with 1000 replications. The code to replicate this simulation is given in Supplemental material Annex 1.

## Results

### Sample characteristics

We recruited 295 patients of which 113 (38%) were female and 182 (62%) male. The mean age was 37 years (standard deviation 25). A total of 35 (12%) had previously been screened with negative results. Among the participants, 94 (32%) had no education, 25 (8%) had been to a madrassa, 41 (14%) to primary school without completing it, 24 (8%) completed primary school, 43 (15%) completed middle school, 14 (5%) went to secondary school without completing it, 41 (14%) completed secondary school, and 13 (4%) had education after secondary school. When asked to read the test sentence, 2 (1%) participants refused, 95 (32%) were unable to read any words, 15 (5%) were only able to read a few words, 45 (15%) were able to read committing 1 or 2 mistakes, and 138 (47%) made no mistakes.

### Performance of the HCVST

Out of 295 participants, 10 (3%) did not correctly interpret the test result and 8 (3%) were not able to complete the test. Of these 8 people, 3 were among those who also reported the test result incorrectly. There were no invalid tests. This meant a total of 15 tests were considered “unsuccessful” and 280 were “successful” based on our criteria, with a success ratio of 95% (95% confidence interval (95% CI) 92–97%). The inter-reader concordance was 96% (95% CI 94–98%), with a Cohen’s kappa value of 0.89. The 10 people who incorrectly interpreted the test result all reported that the test was negative when the study technician reported it as positive. The negative and positive percent agreement values were 100% (95% CI 98–100%) and 84% (95% CI 72–92%), respectively (Table 1).

**Table 1:** Two-by-two table comparing results of HCVST (OraSure Technologies, USA) read by a lay user and a trained technician in a hepatitis C clinic, Karachi, Pakistan, April-June 2023

**HCVST result read by lay user**	**HCVST result read by a trained technician**		
	Negative	Positive	Total
Negative	233	10	243
Positive	0	52	52
Total	233	62	295

When HCVST results reported by participants were compared with results of the second RDT performed on a finger prick sample by the laboratory technician, the inter-operator concordance was 93% (95% CI: 89–95%), with a Cohen’s kappa value of 0.78 (Table 2). The negative and positive percent agreement were 100% (95% CI 98 – 100%) and 70% (95% CI 58 – 80), respectively. A total of 22 patients reporting that they we negative on the oral HCVST were positive on the blood-based RDT (Table 2). Ten (45%) of these 22 HCVST results were reported by the participant as negative but were positive when the test was re-read by the study technician. Twelve (55%) of the 22 HCVSTs were reported as negative by both participant and study technician. Taking only these 12, the positive percent agreement of saliva-based HCVST and the blood-based RDT performed by the laboratory technician was 84% (95% CI 73 – 91).

**Table 2:** Two-by-two table comparing results of saliva-based HCVST (OraSure Technologies, USA) performed by a lay user and results of a blood-based rapid test (Abbott Diagnostics Korea Inc, South Korea) performed by trained technician in a hepatitis C clinic, Karachi, Pakistan, April-June 2023

**HCVST performed by lay user**	**RDT performed by trained technician**		
	**Negative**	**Positive**	**Total**
Negative	221	22	243
Positive	0	52	52
Total	221	74	295

Table 3 shows the number of patients who received verbal assistance for each step of the self-test procedure. For 71% of the individuals, the laboratory technician had to remind the participants to check the time using a clock positioned centrally on the wall so that they could correctly follow the waiting time before test reading. The other step that most frequently required verbal assistance (52% of participants) was the positioning of the buffer on the stand. Overall, 38 (13%) people performed the HCVST without verbal assistance, 67 (23%) needed verbal assistance in one step, 70 (24%) in two and 120 (40%) in three or more.

**Table 3:** Number and proportion of 295 participants who requested verbal assistance from a trained technician for each step in a HCVST performed in Karachi, Pakistan, April-June 2023

Step of the HCVST	With verbal assistance	
	**N**	**(%)**
**Remove buffer tube**	30	(10)
**Open buffer**	115	(39)
**Add buffer to stand**	154	(52)
**Open test kit and remove test**	16	(5)
**Swab mouth**	51	(17)
**Put test in buffer**	41	(14)
**Check time**	209	(71)
**Interpreting test result**	84	(28)

A total of 232 (79%) participants were reported by the observer to have correctly completely swabbed the upper and lower part of the gum; 60 (20%) completely swabbed the upper part of the gum but incompletely swabbed the lower part of the gum; one (0.3%) completely swabbed the lower part of the gum but incompletely swabbed the upper part of the gum; and two (0.7%) incompletely swabbed the upper and lower part of the gum.

According to the observation of the laboratory technician, 85 (29%) people had no difficulties in performing the test, while 82 (28%) had as the main difficulty opening the buffer, 80 (27%) adding the buffer on the stand, 40 (14%) collecting saliva sample and the remaining 8 (2%) in other steps.

### Participant perceptions of HCVST acceptability

Most participants reported that they felt confident that they had done everything (24; 8%) or most things (245; 83%) in the HCVST procedure right, while 26 (9%) thought they had done several or most things wrong. The two steps that participants reported they found most difficult were opening the buffer and adding the buffer to the stand, with 11% and 6% of participants reporting these steps as difficult, respectively (Fig. 1).

Most participants (291, 99%) stated that they could perform the HCVST on their own at home without help from anyone. All participants (295, 100%) responded that they would use HCVST again in the future and would recommend it to friends and family members. When asked: "What do you think is the main advantage of HCVST?", 221 (75%) replied that this way they do not have to access a clinic, 63 (21%) they can do the test themselves at any time, 9 (3%) the greater privacy and 2 (1%) other advantages. When asked: "What do you think is the main disadvantage of HCVST?", 230 (78%) replied that there are no disadvantages, 40 (14%) were not sure, 10 (4%) the low confidence in results, 10 (3%) the absence of counselling and 2 (1%) the difficulty of performing the test alone. Most participants (207; 70%) reported that if they had to repeat the test at home, they would prefer to use a saliva-based self-test, while 87 (30%) would prefer a blood-based self-test. One individual did not know. Most respondents (284, 96%) when asked if they were to test in the future would prefer HCVST over a test performed in a clinic.

## Discussion

Increasing access to HCV testing among high-prevalence groups in LMICs will be essential to achieve WHO viral hepatitis elimination goals by 2030. Recently published reports showed high usability and acceptability of HCV self-testing in the general population in Egypt, South Africa, USA and Malaysia [14–16] as well as in high-risk populations in China, UK, Malaysia, Kenya, Rwanda and Kyrgyzstan (16–18,20–22). We conducted the first study to assess the usability and acceptability of HCVST among a high HCV-seroprevalence population living in an informal settlement in Pakistan. Acceptability was very high, with all participants agreeing that they would use the test again in the future and recommend it to family and friends. The majority also reported they would prefer HCVST to a clinic-based test. The success ratio was also high, suggesting HCVST could be a useful addition to testing performed by trained staff in this setting. However, we also found moderately low positive percent agreement when comparing reported HCVST result with the result of an RDT performed by trained personal on a finger prick sample. Further studies are required to fully evaluate HCVST accuracy, but these findings highlight the risks for false negative results with saliva based HCVST in this setting.

Our results show that critical faults in performing the HCVST procedure were uncommon and most participants were able to obtain and interpret results accurately. However, most participants required verbal assistance in the implementation of the HCVST. Our study protocol was that this assistance would be offered only if the participant was considered unable to proceed to the next stage without assistance. It is unclear whether the assistance was always necessary to successfully complete the HCVST procedure or whether requests for support were made more likely by the presence of the study technician observing the HCVST. High demand for assistance in the self-testing process was observed in a similar study conducted among PWID in Kenya [18]. In view of this high demand for verbal assistance, a hotline could be implemented to answer questions from people who are performing HCVST at home. Such hotlines may be hard to sustain for small-scale projects using HCVST but may be more feasible if HCVST could be integrated into regional or national screening programmes.

The most common step that required verbal assistance among the participants in this study was practising good timekeeping. This was also reported as the main difficulty in studies done in Malaysia, Kenya and China [16–18]. This critical step could affect the sensitivity of HCVST, as reading the test before the recommended reading window may lead to invalid or false negative results. Such mistakes might be prevented by further optimizing the IFU to include stronger emphasis on time keeping and providing additional support material such as video instructions accessible on a smartphone. The use of a smartphone application with timer function during HCVST may also guide users to perform time keeping steps accurately. We do not have data on the level of smartphone ownership in Machar Colony and the use of phone-based applications in low-income settings should take care not to further marginalise the poorest from HCV screening. The two other steps in which verbal assistance was most frequently required were opening the buffer and placing the buffer in the stand. Previous studies on usability of HCVST also reported difficulties with these two steps [14, 17]. These issues could potentially be resolved through the design of specific self-test kits that are easier for lay users to use, for example by integrating simpler buffer delivery systems that do not require the user to open buffer tubes or place buffer tubes in stands.

Despite the high number of individuals who encountered difficulties and needed verbal assistance while conducting the test, concordance measures were high. The concordance between lay and trained readers was higher than that observed in the pilot study of HCVST in Egypt (97% vs. 86%) and similar to that observed in a study in Malaysia (96% vs. 97%) [15, 16]. The same was observed for inter-operator concordance and was higher than that reported in Egypt (93% vs. 89%) and similar to that reported in Malaysia (93% vs. 91%) (15,16).

It is important to note that the high overall concordance measures we observed were driven in large part by the perfect agreement between negative results read by the lay and trained user and between negative HCVST results and the results of the second RDT. When considering only positive test agreement, errors were more likely. We did not formally evaluate HCVST accuracy against a reference standard serological test for HCV exposure and therefore do not report sensitivity or specificity measures here. However, the RDT performed by a trained user on a finger prick sample is considered to be highly accurate, with the manufacturer reporting a sensitivity and specificity of 99.3% and 98.1%, respectively [28]. Taking this RDT result to reflect serostatus, our data indicate that around 30% of HCVSTs performed among people with seropositive status were falsely negative. The confidence intervals around the positive percent agreement estimate were wide, reflecting substantial uncertainty. However, these results raise concerns that people with HCV infection may be missed if HCVST is used unsupervised in this setting. Around 45% of the false negatives we identified were due to the participant incorrectly interpreting the HCVST result as negative when the trained technician read the same test as positive. Correct interpretation of test results is particularly hard to convey using pictorial instructions for illiterate users. Clear instructions on reading test results and interpreting test bands should be given verbally or through the use of video-based media when tests are provided in settings with low literacy. The remaining false negative HCVST results were read as negative by both the participant and trained technician. The reason for the high error ratio here is unclear but may be due in part to poor swabbing technique and insufficient saliva on the swab. Swabbing was reported as a common cause of difficulty among participants by the study technician observing the HCVST.

The level of acceptability we found in this study was very high and is in line with what has been reported in the literature [14, 17]. Despite the high acceptability of the saliva-based HCVST used in this study, 30% of the participants expressed a preference for blood-based HCVST. The reason for this preference was not investigated but research on HIVST reported some users perceive the blood-based test to be more accurate, despite being potentially harder to perform [12, 29]. The WHO guidance on HCVST encourages national programmes to offer a choice in the type of self-test kits provided and sample types collected, promote diversity of providers and address the preferences of different population groups as it can increase acceptance [12].

There were several limitations that should be considered when interpreting our findings. First, the number of individuals excluded or refused to participate was not systematically recorded, preventing an assessment of this potential bias. Second, HCVST is intended to be used privately and the presence of an observer in the room during the self-testing procedure may have resulted in an abnormally high rate of requests for assistance. This has the potential to bias the results to higher success ratio. Third, this study was limited to oral fluid testing and was not able to assess acceptability and usability of other testing modalities, including finger stick blood testing. Given the preference we observed for blood-based HCVST among a large number of our participants, usability of self-testing using a finger prick sample would be a valuable focus for future work in Pakistan. Fourth, the HCVST kits used in this study were only for research use, and the packaging and instructions for use may be different from a product intended for self-testing. Finally, the study was conducted in a clinic-based setting rather than in the community. This may overestimate usability, acceptability and the success ratio due to differences in characteristics of clinic attendees compared with people who are less likely to attend the clinic. We did not formally evaluate this, but this may include differences such as level of literacy.

Our findings have several implications for operationalisation of HCVST. First, there is a need to minimize errors and difficulties related to self-testing by simplifying tests and test procedures, improving test devices to address issues such as ease of opening and use of the buffer, optimizing IFU, and providing support tools such as the use of hotlines. This could also include the use of instructional videos for some individuals or populations, such as those with low literacy levels. Such tools have been developed and successfully implemented during the roll-out of HIVST [7, 30]. Secondly, studies are needed to compare the HCVST approach with other community- and facility-based HCV testing to identify the optimal positioning of self-testing for promoting access to testing and treatment. The very high acceptability of HCVST observed in this setting suggests it can play an important role in reducing undiagnosed infections and improving access to care services [31]. However, our findings also highlight the risks for false negative results for HCVST in the hands of lay users. Future studies should formally estimate the diagnostic sensitivity and specificity of unsupervised HCVST. Such research would help better evaluate the balance between improved access to screening for HCV using HCVST with the potential for cases to be missed.

## Conclusion

Our study demonstrated that saliva-based HCV self-testing has very high acceptability in this setting with a high HCV prevalence and low levels of literacy. However, we found that users requested high levels of support during implementation and faced particular challenges around handling the buffer and in time keeping. Innovative approaches that can improve comprehension of self-test procedures, as well as the availability of self-test kits that are easier to use, are expected to increase HCVST usability and accuracy, particularly in settings with low literacy. Further work is required to explore the operationalisation of HCVST in informal settlements, such as Machar Colony, including when and to whom HCVST should be offered. While the high acceptability demonstrated in this study suggests HCVST can play an important role in increasing screening access and uptake, the low positive percent agreement we observed when comparing results of HCVST with the results of a test performed by a laboratory technician highlights the risks for cases to be missed when these tests are used by lay users. These results suggest screening tests performed by health workers should be preferred over HCVST in situations where people can be reached by health worker testing.

## Supplementary Information

Supplementary Material 1.

## Data Availability

The datasets used and analysed during the current study are available from Médecins Sans Frontières, Operational Centre Brussels, on reasonable request.

## Abbreviations

HCV: Hepatitis C virus,
HCVST: HCV-antibody self-testing,
LMICs: Low- and middle-income countries
DAA: Direct-acting antiviral
WHO: World Health Organization
RDT: Rapid diagnostic tests
HIV: Human immunodeficiency virus
MSF: Médecins Sans Frontières
CHC: Chronic hepatitis C
PCR: Polymerase chain reaction
IFU: Instructions for use
