# Supplementary material for: Usability and acceptability of self-testing for hepatitis C virus exposure in a high-prevalence urban informal settlement in Karachi, Pakistan
Source: BMC Infect Dis. 2024 Sep 27;24:1054. doi: 10.1186/s12879-024-09925-6 (PMC11428378; doi:10.1186/s12879-024-09925-6)
Supplement: Supplementary file 1 — Supplementary Material 1. [file 12879_2024_9925_MOESM1_ESM.docx]

Supplemental material

**R code for sample size estimates**

p = 0.87 ### lowest expected success ratio of worst performing hcvst samplesize = 200

nn = 100000 #### a large population from which we draw our sample

dta = as.data.frame(matrix(NA, nrow = nn, ncol=1)) # data frame to fill

dta[,1] = c(1:n) #### row id

names(dta) = "id"

dta$hcvst <- rbinom(nrow(dta), 1, p) # for each individual, success of hcvst given p

s = 2000 #### number of simulations

B = as.data.frame(matrix(NA, nrow = s, ncol = 5))

for (i in 1:s){

print(i)

sample = sample(1:nn, samplesize, replace=F)

newdata = dta[sample,]

B[i,1] = mean(newdata$hcvst)

x = prop.test(round(sum(newdata$hcvst), 0), nrow(newdata), conf.level = 0.9)

xx = binom.test(round(sum(newdata$hcvst), 0), nrow(newdata), 0.8, conf.level = 0.95,alternative = "greater")

B[i,2] =  x$conf.int[1]

B[i,3] =  x$conf.int[2]

B[i,4] =  ifelse(x$conf.int[1]> 0.8, 1, 0)

B[i,5] =  ifelse(xx$p.value < 0.05, 1, 0)}

summary(B)

Figure 1 Pictorial instructions for use (IFU) that were purposively developed for this study. They are complemented by short sentences in Urdu, the national language. In blue is the English translation.


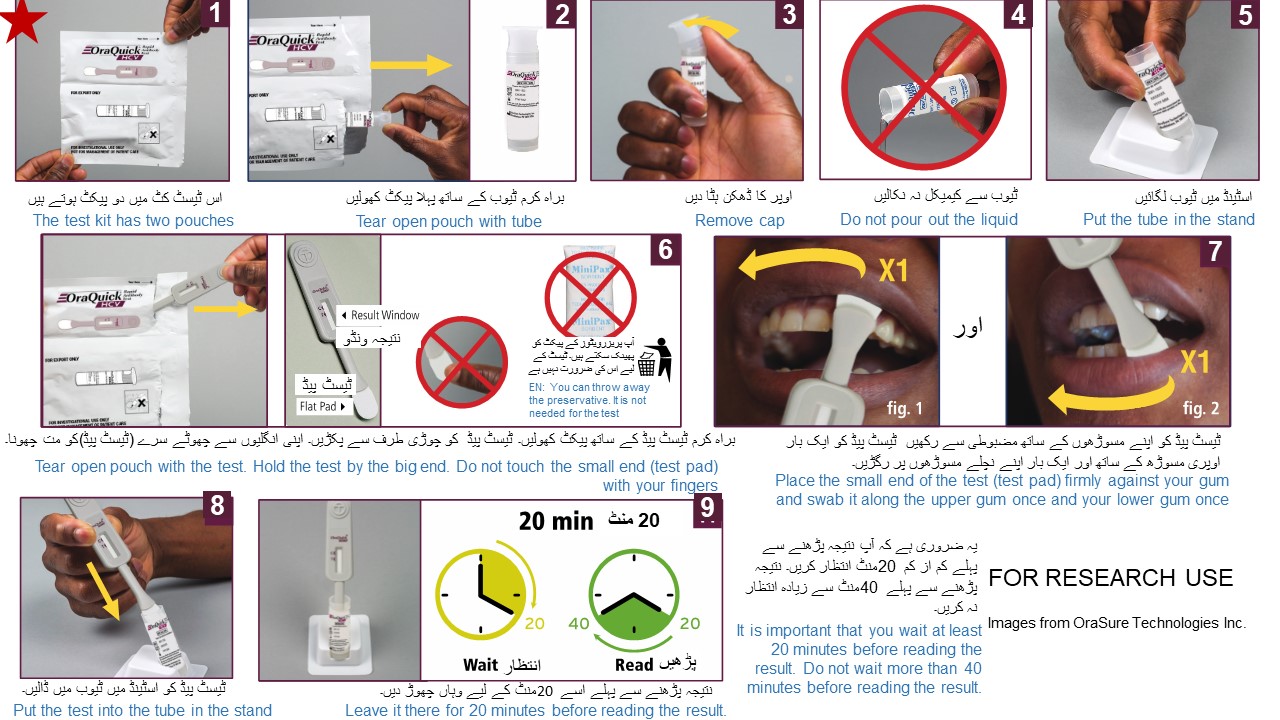


Figure 2 Pictorial instructions for result interpretation. They are complemented by short sentences in Urdu, the national language. In blue is the English translation.
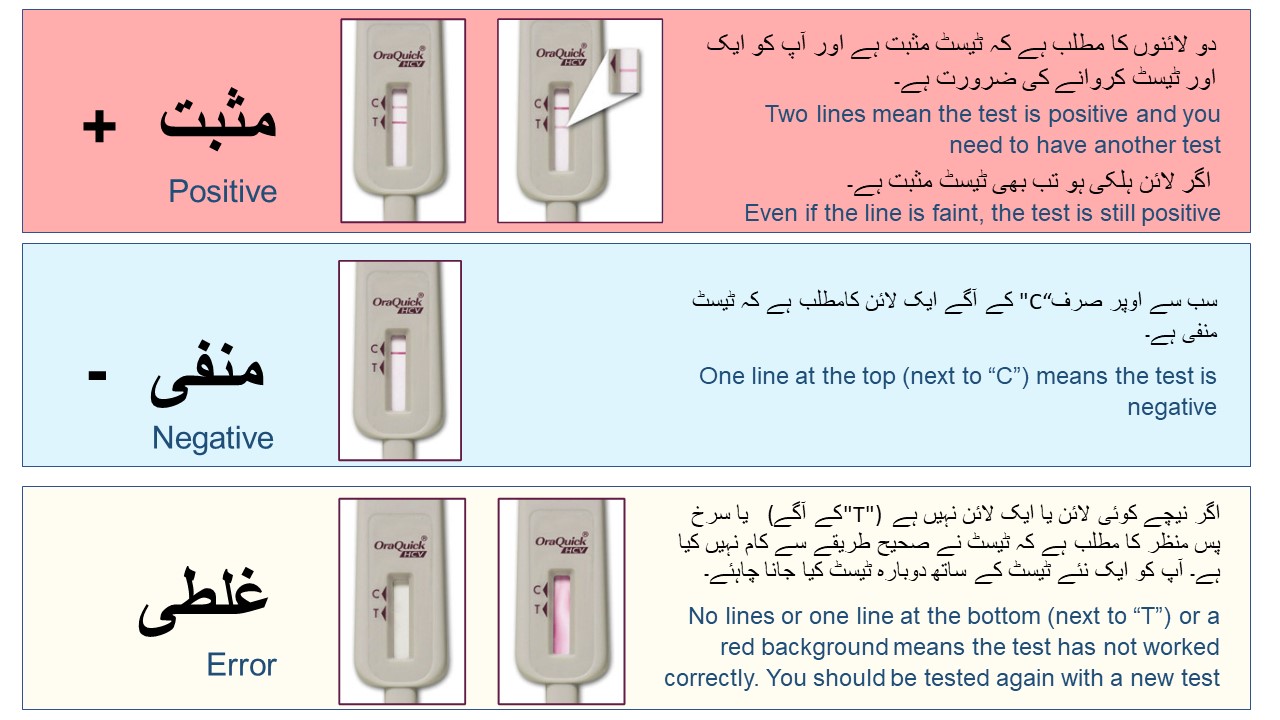


### Annex 1 Self-test questionnaire

1. Individual ID:
2. Have you been tested for HCV before?

$\square$ Yes; $\square$ No; $\square$ Don’t Know

1. If yes, what was the year of your last test?
2. Sex:

$\square$ Male; $\square$ Female

1. Age in years:
2. What level of schooling did you reach?

$\square$ No schooling; $\square$ Madrassa; $\square$ Some primary school; $\square$ Completed primary school; $\square$ Middle school; $\square$ Some secondary school; $\square$ Completed secondary school; $\square$ Post secondary course (university, diploma etc); $\square$ Prefer not to say

1. Are you able to read this line of Urdu out loud to me: *“Today we are testing for a disease of the liver that can affect people of any age.”*?

$\square$ Read aloud with no errors; $\square$ Read aloud with errors on one or two words; $\square$ Only a few words correctly read aloud; $\square$ Cannot read any words aloud; $\square$ Reports unwilling to read aloud.

**Saliva-based test**

1. **Checklist** (*record when observed*):

$\square$ Sanitised hands without intervention; $\square$ Opened box; $\square$ Read instructions; $\square$ Removed test from pouch; $\square$ Removed cap from vial; $\square$ Placed vial in stand; $\square$ Completely swabbed around upper part of gum; $\square$ Completely swabbed around lower part of gum; $\square$ Inserted device into the buffer solution; $\square$ Checked time at start of test; $\square$ Test fluid ran across strip; $\square$ Discarded test in waste bag; $\square$ Put cap on vial and discarded in waste bag

1. **Faults** (*record when observed*):

$\square$ Touched swab with fingers; $\square$ Swabbed too deep in mouth (behind teeth); $\square$ Limited swabbing upper mouth; $\square$ Limited swabbing upper mouth; $\square$ Used equipment not put in waste bags; $\square$ Dropped test kit on floor; $\square$ Dropped vial on floor; $\square$ Difficulty adding vial to stand; $\square$ Spilled buffer in work site; $\square$ No mistakes observed; $\square$ Other (specify)

1. What single thing would you say the participant found most difficult (*select one*):

$\square$ Opening test kit; $\square$ Opening vial; $\square$ Adding vial to test stand; $\square$ Collecting saliva sample; $\square$ Adding test to buffer; $\square$ They didn’t seem to face any difficulties; $\square$ Other (specify)

1. Did you have to provide additional verbal assistance in any of the following steps (*select all that apply*):

$\square$ Familiarising with instructions; $\square$ Identifying test kit; $\square$ Identifying vial; $\square$ Identifying rack; $\square$ Opening test kit; $\square$ Opening vial; $\square$ Collecting saliva sample; $\square$ Adding test kit to buffer; $\square$ Managing waste; $\square$ Other (specify)

1. Did you have to provide direct assistance in any of the following steps (*select all that apply*):

$\square$ Collecting saliva sample; $\square$ Adding sample to buffer; $\square$ Other (specify)

*To the participant:*

1. How confident do you feel that you performed the test correctly (*select one*)?

$\square$ I feel very confident that I did everything right;$\square$ I think I did most things right;$\square$ I think I got several things wrong;$\square$ I think I got most things wrong

1. Do you think the written instructions are easy or difficult to understand (*select one*)?

$\square$ Very easy; $\square$ Easy; $\square$ Difficult; $\square$ Very difficult; $\square$ Didn’t read them

1. Do you have any specific recommendations on how we can improve the written instructions?
2. Do you think the pictures are easy or difficult to follow (*select one*)?

$\square$ Very easy; $\square$ Easy; $\square$ Difficult; $\square$ Very difficult; $\square$ Didn’t read them

1. Do you have any specific recommendations on how we can improve the picture-based instructions?
2. How difficult or easy did you find each of the following steps:
3. Opening test kit (*select one*):

$\square$ Very difficult; $\square$ Difficult; $\square$ Slightly difficult; $\square$ Slightly easy; $\square$ Easy; $\square$ Very easy

1. Opening the vial (*select one*):

$\square$ Very difficult; $\square$ Difficult; $\square$ Slightly difficult; $\square$ Slightly easy; $\square$ Easy; $\square$ Very easy

1. Adding vial to stand (*select one*):

$\square$ Very difficult; $\square$ Difficult; $\square$ Slightly difficult; $\square$ Slightly easy; $\square$ Easy; $\square$ Very easy

1. Collecting saliva sample (*select one*):

$\square$ Very difficult; $\square$ Difficult; $\square$ Slightly difficult; $\square$ Slightly easy; $\square$ Easy; $\square$ Very easy

1. Adding test kit to vial (*select one*):

$\square$ Very difficult; $\square$ Difficult; $\square$ Slightly difficult; $\square$ Slightly easy; $\square$ Easy; $\square$ Very easy

*Questions to participant after saliva HCVST test performed*

1. Overall, would you describe this test as difficult or easy to perform (*select one*)?

$\square$ Very difficult; $\square$ Difficult; $\square$ Slightly difficult; $\square$ Slightly easy; $\square$ Easy; $\square$ Very easy

1. Do you think you could perform this test by yourself at home without anyone there to support you?

$\square$ Yes $\square$ No $\square$ Don’t know

1. Do you think you could read the results alone?

$\square$ Yes; $\square$ No; $\square$ Don’t know

1. If the test was free of charge and could be collected from the clinic or delivered to your home, do you think you would use self-testing in the future? $\square$ Yes; $\square$ No; $\square$ Don’t know
2. Would you recommend self-testing to a friend or family member?

$\square$ Yes; $\square$ No; $\square$ Don’t know *(If no or don’t know, why?)*

1. What do you think are the advantages of self-testing for hepatitis C?

$\square$ Privacy; $\square$ No need to come to a clinic; $\square$ I can test myself anytime; $\square$ Not sure; $\square$ Other (specify)

1. Which are the disadvantages of self-testing for hepatitis C?

$\square$ Difficult to perform; $\square$ Low confidence in results; $\square$ No counselling; $\square$ Don’t know; $\square$ Other (specify)

1. If you could do a test at home that was similar to the one you performed today but used a saliva sample from your mouth [blood sample from a small prick to your finger] rather than blood [saliva], would that be preferable?

$\square$ Yes; $\square$ No; $\square$ Don’t know

1. If you were tested for HCV in the future, which of the following would be your preference for where and how the test is done:

$\square$ At home by myself; $\square$ At home by a family member or friend; $\square$ At home by a visiting health care worker; $\square$ By myself in a private place outside my home; $\square$ At the MSF clinic or another clinic close to my home; $\square$ Other (specify)

*Reading results*

1. What result does the participant report?

$\square$ Positive; $\square$ Negative; $\square$ Invalid

1. What is the correct result?

$\square$ Positive; $\square$ Negative; $\square$ Invalid

What is the result of the clinic test (performed by MSF staff)?

$\square$ Positive; $\square$ Negative; $\square$ Invalid
